# Combined Extracts of Herba Epimedii and Fructus Ligustri Lucidi Rebalance Bone Remodeling in Ovariectomized Rats

**DOI:** 10.1155/2019/1596951

**Published:** 2019-02-13

**Authors:** Yuheng Chen, Xiaoxi Li, Xiufeng Tang, Yingying Gao, Ping Yu, Liping Xu, Renhui Liu

**Affiliations:** School of Traditional Chinese Medicine, Capital Medical University and Beijing Key Lab of TCM Collateral Disease Theory Research, No. 10 Xitoutiao, Youanmenwai, Fengtai District, Beijing 100069, China

## Abstract

This study aimed to investigate the osteoprotective effect and the possible molecular mechanisms of the combined extracts of Herba Epimedii and Fructus Ligustri Lucidi on postmenopausal osteoporosis (PMOP). Forty-eight female SD rats were sham-operated (Sham,* n* = 8) or ovariectomized (OVX,* n* = 40). Then after a week, OVX rats were divided randomly into five groups (*n *= 8 in each group): OVX, extracts of Herba Epimedii (HE, 0.35 g/kg), extracts of Fructus Ligustri Lucidi (FLL, 0.35 g/kg), combined extracts of HE and FLL (HE & FLL, 0.20 g/kg HE plus 0.15 g/kg FLL), and Raloxifene hydrochloride (RH, 6.25 mg/kg) groups. All groups were administered once daily for 12 weeks. Indicators related to bone remodeling were detected, including estradiol (E_2_), bone mineral density (BMD), maximal load, ultimate deflection, micro-CT properties, tartrate-resistant acid phosphatase (TRACP) and alkaline phosphatase (ALP) levels in serum and bone, and the protein and mRNA expression of bone turnover markers (RANKL, M-CSF, Wnt5a, Atp6v0d2, OPG, IGF-1, TGF-*β*1, and Bmp-2). Results showed that the combined extracts could increase serum E_2_ levels and BMD, enhance bone strength, reserve bone microstructure degeneration, promote bone formation, and inhibit bone resorption through upregulating the mRNA and protein expression of OPG, IGF-1, TGF-*β*1, and Bmp-2, while downregulating RANKL, M-CSF, Wnt5a, and Atp6v0d2. These findings demonstrated that the combined extracts of Herba Epimedii and Fructus Ligustri Lucidi with bone protective effects on OVX rats might be an alternative medicine for the treatment of PMOP.

## 1. Introduction

Postmenopausal osteoporosis (PMOP) is a serious health problem related to postmenopausal women, which is characterized by skeletal fragility and microarchitecture deterioration [1]. The clinical management of PMOP relies on calcium supplement and hormone replacement therapy (HRT), including calcitonin, estrogen, and selective estrogen receptor modulator. However, their side-effects, such as gastrointestinal side effects, coronary heart disease, and high risk of gynecological cancer, severely limit their long-term clinical application [2, 3]. Therefore, it is necessary to develop effective new anti-PMOP drugs.

In recent years, growing interests were placed into the treatment of PMOP with plant-based therapies including traditional Chinese medicines (TCM). According to the theory of TCM that the kidney being in charge of bone is responsible for bone growth and development, Herba Epimedii (HE) and Fructus Ligustri Lucidi (FLL), approved as tonifying-kidney agents by TCM doctor more than 2000 years ago, are often used to treat orthopedic related diseases. We previously demonstrated that the combined extracts of HE and FLL had an osteoprotective effect in osteoporosis rat models induced by retinoic acid and glucocorticoid [4–6]. In addition, many studies had shown that the total flavonoids of HE [7, 8] and the total iridoids and flavonoids of FLL [9, 10] had an antiosteoporosis actions. But the effects of the combined herbs on PMOP were still unclear.

The basic treatment of the combined HE with FLL for osteoporosis is to adjust the balance between yin and yang and invigorate the kidney to strengthen bone. The balance between yin and yang in bone is equal to the homeostasis of bone remolding. Under physiological conditions, the lacunas by bone resorption are filled by the same amount of new bone formed by osteoblasts (OBs), which makes the two closely linked in time and space. This coupling relationship of osteoclasts (OCs) and OBs is the key to maintaining the homeostasis of bone remolding [11]. The pathogenesis of PMOP is mainly the negative balance of bone remolding caused by estrogen deficiency [12], so restoring the balance between bone formation and resorption is the core of treatment. The purpose of this study was to investigate whether combined extracts of HE and FLL could have an antiosteoporosis effect on PMOP and explore the mechanism of rebalancing bone remolding.

## 2. Materials and Methods

### 2.1. Preparations of Herbal Materials

Extracts of Herba Epimedii (HE) and Fructus Ligustri Lucidi (FLL) were purchased from Xian Kangwei Bioengineering Co., Ltd. (Xian, China). The extracts of HE and FLL were prepared by reference to previous reports [13]. Both the yields of HE and FLL extracts were 10%. The detection results of HE and FLL components were in compliance with the 2015 Chinese Pharmacopoeia phytochemical test regulations.

### 2.2. Animals

Female Sprague-Dawley rats (8-month-old; weight 390 ± 10 g) were purchased from Beijing Vital River Laboratory Animal Technology Co. Ltd. (Beijing, China). All experimental protocols were approved by the Ethics Committee of Capital Medical University (No. AEEI-2016-178). The rats were housed in stainless cages (2 rats* per* cage) under normal control conditions, and standard laboratory light, tap water, and diet were provided throughout the experiments.

The rats were randomly divided into Sham group (*n* = 8) and ovariectomized (OVX) group (*n* = 40). Rats in Sham or OVX group were subjected to either a sham surgery or bilateral ovariectomy as described previously [14]. One week after recovering from surgery, the OVX rats were randomly divided into five groups (8 rats* per* group): OVX, extracts of Herba Epimedii (HE, 0.35 g/kg/day), extracts of Fructus Ligustri Lucidi (FLL, 0.35 g/kg/day), combined extracts of HE and FLL (HE & FLL, HE 0.20 g/kg/day plus FLL 0.15 g/kg/day), and Raloxifene hydrochloride (RH, 6.25 mg/kg/day). All groups were administered once daily for 12 weeks and then sacrificed.

### 2.3. Bone Mineral Density (BMD) Measurement

BMD in the right femurs were detected using a dual-energy X-ray absorptiometry system (DEXA, Lexxos-2000, Medlink, France) according to our previous research [15]. The scanning positions included the whole femur and the femur head.

### 2.4. Bone Biomechanical Testing

The biomechanical properties of the right femur were assessed using a 3-point bending test on a WD-1 Universal Testing Apparatus (KeXin Testing Machine Co., Ltd., Changchun, China) according to our previous research [15]. Loading-displacement curves were recorded online and analyzed to determine the bone biomechanical properties of ultimate load and displacement at ultimate.

### 2.5. Micro-Computerized Tomography

The entire scan length was set for 5 mm from top of left femur to proximal direction of femur in a spatial resolution of 17 *μ*m per voxel with a 3072 × 2048 image matrix by micro-CT system (Inveon, Siemens, Germany). Then we construct the microscopic 3D structure of the trabecular bone. Bone tissue volume fraction (Bone Volume/Total Volume, BV/TV), bone surface fraction (Bone Surface/Bone Volume, BS/BV), trabecular number (Tb. N), trabecular thickness (Tb. Th), trabecular separation (Tb. Sp), and cortical wall thickness (CWT) were measured separately using built-in software.

### 2.6. ALP and TRACP Testing

Serum bone specific alkaline phosphatase (bALP) and tartrate-resistant acid phosphatase (TRACP) were measured by ELISA kits (Blue Gene Biotech Co., Ltd, Shanghai, China) according to the protocols provided by manufactures with a SpectraMax Plus 384 Microplate Reader (Molecular Devices, Sunnyvale, CA, USA). The sections of the decalcified tibias were stained histochemically for ALP and TRACP activity as an OB and OC marker, respectively, using ALP or TRACP stain kit (Nanjing Jiancheng Bio, Nanjing, China) according to the manufacturer's recommendations. The stained slides were observed using an image analyzing computer system (NIS-Elements BR 3.2, Nikon, Japan) linked to a light microscope (Nikon Eclipse 80i, Tokyo, Japan). The positive reaction of ALP staining showed gray-black particles or blocky precipitates, and TRACP staining showed dark red, localizing the cytoplasm. Integral optical density (IOD) of ALP or OC number in TRACP-stained sections was determined.

### 2.7. Immunofluorescence (IF) Staining

The tibia was fixed in a 10% formaldehyde solution and then used to make a 5 *μ*m thick longitudinal section. The antigen of the sections was repaired with citrate buffer at pH 6.0 after gradient dewaxing and then blocked with goat serum. The sections were incubated with primary antibodies against the receptor activator of nuclear factor-kappa B ligand (RANKL), the macrophage colony-stimulating factor (M-CSF), Wnt5a, the d2 isoform of vacuolar (H^+^) ATPase (v-ATPase) V0 domain (Atp6v0d2), osteoprotegerin (OPG), insulin-like growth factor-1 (IGF-1), transforming growth factor-*β*1 (TGF-*β*1), and bone morphogenetic protein-2 (Bmp-2) (1:500), respectively, at 4°C overnight. Subsequently, the sections were washed with PBS and incubated with corresponding secondary antibody (1:200, Zhong Shan Golden Bridge Biotechnology, Beijing, China) at room temperature for 1 h. The sections were then costained with DAPI and then observed. We used a Nikon ECLIPSE 80i biomicroscope to magnify 200 × and selected 3 random regions for each section. IOD were measured using NIS-Elements BR 3.2 image analysis system (Nikon, Japan). Then calculate the average of the three regions as the final result of the sample.

### 2.8. Analysis of mRNA Expression by qRT-PCR

After the bone tissue was ground in liquid nitrogen, total RNA was extracted with an RNAprep pure tissue kit, followed by total RNA reverse transcription using a FastQuant RT kit (With gDNase), and finally SuperReal PreMix Plus (SYBR Green) and Bio-Rad PCR cycler (CFX96 Real-Time System) for real-time quantitative analysis. The PCR total reaction system was selected to have a volume of 20 *μ*L per well to make three duplicate wells. The PCR reaction system included 10 *μ*L of 2 × SuperReal PreMix Plus, 0.6 *μ*l of each of the forward and reverse 10 *μ*M primers, 2 *μ*L of H_2_O, and 2.0 *μ*L of cDNA. The PCR reaction cycle was 95°C for 15 minutes, then 40 cycles of 95°C denaturation for 12 seconds, 60°C annealing for 1 minute, and 60°C for 10 seconds. Each sample was normalized using *β*-actin as an internal reference, and the results were statistically analyzed by the 2^-ΔΔCt^ equation. The sequences of primers were shown in Table 1.

### 2.9. Serum Estradiol (E_2_) Testing

Serum E_2_ was measured by ELISA kits (Blue Gene Biotech Co., Ltd, Shanghai, China) according to the protocols provided by manufactures with a SpectraMax Plus 384 Microplate Reader (Molecular Devices, Sunnyvale, CA, USA).

### 2.10. Statistical Analysis

Data were presented as mean ± standard error of mean (SEM). The statistical differences among groups were evaluated using SPSS 24.0 software (SPSS Inc., Chicago, USA) by one-way analysis of variance (ANOVA). The least significant difference (LSD) test when the variances are equal or Tamhane's T2 test when the variances are not equal was used for comparisons between individual groups and to determine which means differed statistically significantly (*P* < 0.05).

## 3. Results

### 3.1. Effects of HE & FLL on BMD and Bone Biomechanical Properties

**Figure 1:**
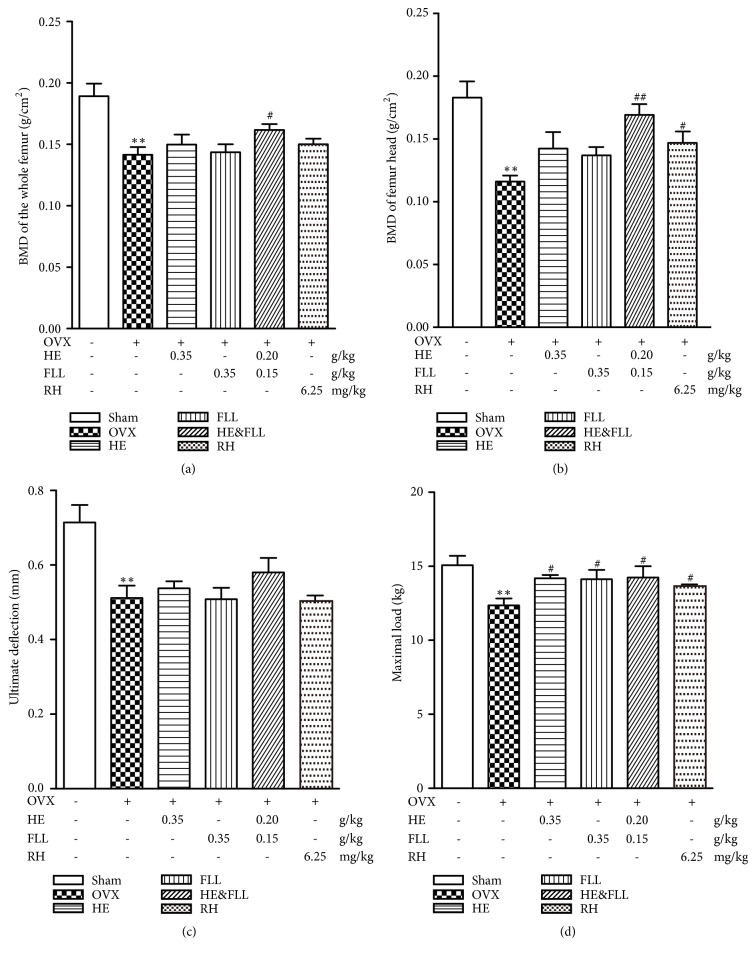
*Effects of HE & FLL on BMD and bone biomechanical properties in OVX rats*. (a-b) BMD was measured by dual-energy X-ray absorptiometry. (c-d) Bone biomechanical properties, including ultimate deflection and maximal load. Data are represented as mean ± SEM,* n* = 6. Note: ^*∗∗*^*P* < 0.01 versus Sham; ^#^*P* < 0.05 and ^##^*P* < 0.01 versus OVX.

The gold indicator for the diagnosis of OP is to detect bone density by dual-energy X-ray absorptiometry (DEXA) [16]. We used DEXA to detect the BMD levels of right femur to determine whether the extracts have anti-PMOP effects. The BMD level in OVX group was obviously lower than that in Sham group (*P* < 0.01). In HE & FLL group, BMD levels of rats in the whole femur and femur head were significantly increased than these in OVX rats (*P* < 0.05 or* P* < 0.01) (Figures 1(a)-1(b)).

The biomechanical integrity of bone is considered as the main factor associated with the risk of fracture [17]. We evaluated the biomechanical state of the bone by observing the maximum displacement of the fracture (maximum load) and the vertical displacement of the fracture surface and the long axis (ultimate deflection) of the right femur. Figures 1(c)-1(d) showed that bilateral OVX could induce significantly reduction in maximal load and ultimate deflection, compared with Sham group (*P* < 0.01). After the treatment, femur maximal load could be obviously improved in all the treatment groups compared with the OVX group (*P* < 0.05). But no significant differences in ultimate deflection were present among HE, FLL, HE & FLL, RH, and OVX groups (*P* > 0.05).

### 3.2. Effects of HE & FLL on Micro-CT

**Figure 2:**
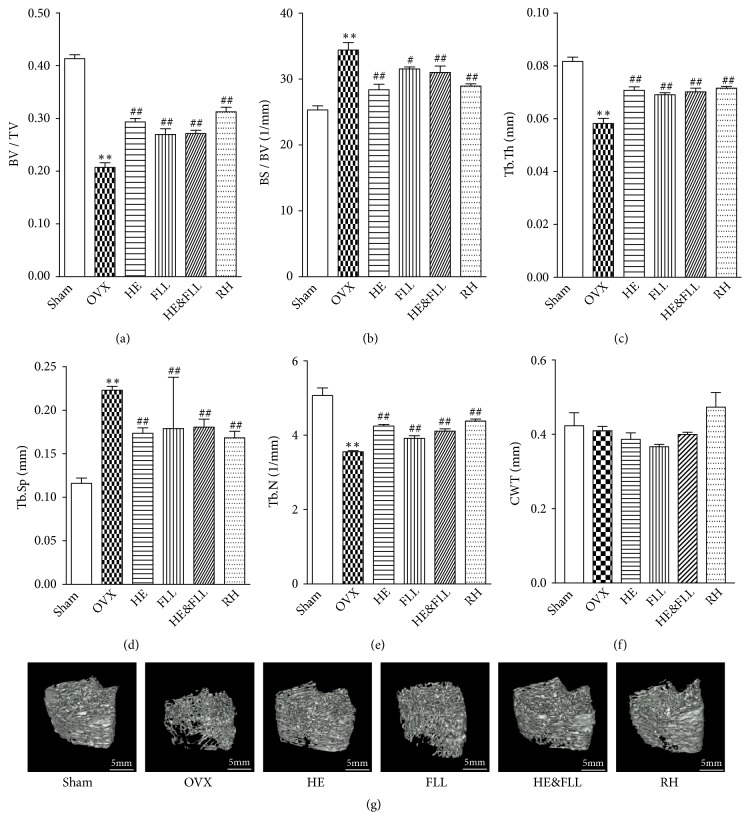
*Effects of HE & FLL on micro-CT in OVX rats*. (a-e) Parameters of trabecular bone microarchitecture by micro-CT. (f) Cortical wall thickness by micro-CT. (g) 3D images of trabecular bone microarchitecture. Data are represented as mean ± SEM,* n* = 3. Note: ^*∗∗*^*P* < 0.01 versus Sham; ^#^*P* < 0.05 and ^##^*P* < 0.01 versus OVX.

The wide application of micro-CT makes the detection of bone microstructure more sensitive and efficient [18].  Figure 2 showed that trabecular bone of Sham group was dense and highly connected, while the trabecular bone of OVX group was significantly less, thinner, and less tightly connected. After treatment, the trabecular bone of the rats was significantly increased, thickened, and more connected. These results showed that compared with Sham rats, Tb.N, Tb.Th, and bone volume fraction of OVX rats were markedly decreased, and Tb.Sp was significantly increased (*P* <0.01). The above situation was alleviated in each treatment group, compared with OVX rats (*P* < 0.05 or* P* < 0.01). There was no significant difference in CTW between groups (*P* > 0.05).

### 3.3. Effects of HE & FLL on Serum E_2_ Levels

**Figure 3:**
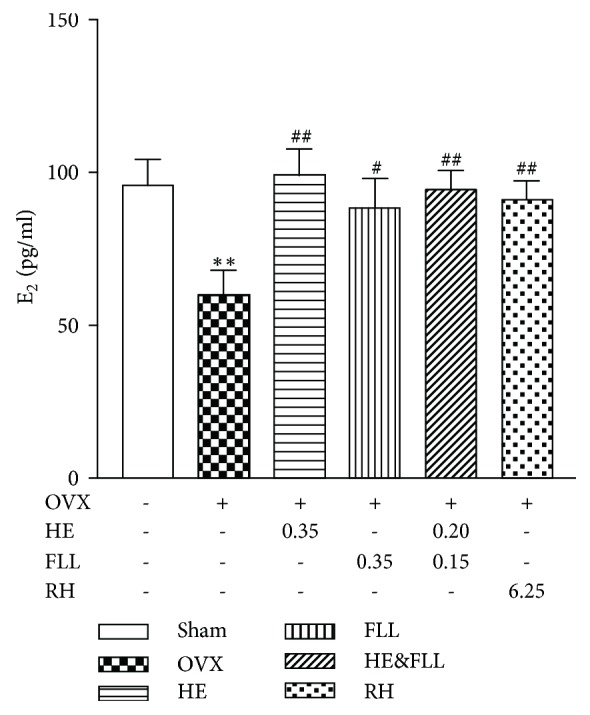
*Effects of HE & FLL on serum E*_2_ *levels*. Data are represented as mean ± SEM,* n* = 8. Note: ^*∗∗*^*P* < 0.01 versus Sham; ^#^*P* < 0.05 and ^##^*P* < 0.01 versus OVX.

Estrogen deficiency is a typical feature of OVX rats. As shown in Figure 3, compared with Sham group, the significant decrease of serum E_2_ levels in OVX rats was found (*P* < 0.01), indicating successful depletion of E_2_ after OVX. After treatment, clear increase of serum E_2_ levels was observed in all treatment groups when compared with OVX group (*P* < 0.05 or* P* < 0.01).

### 3.4. Effects of HE & FLL on TRACP/ALP

**Figure 4:**
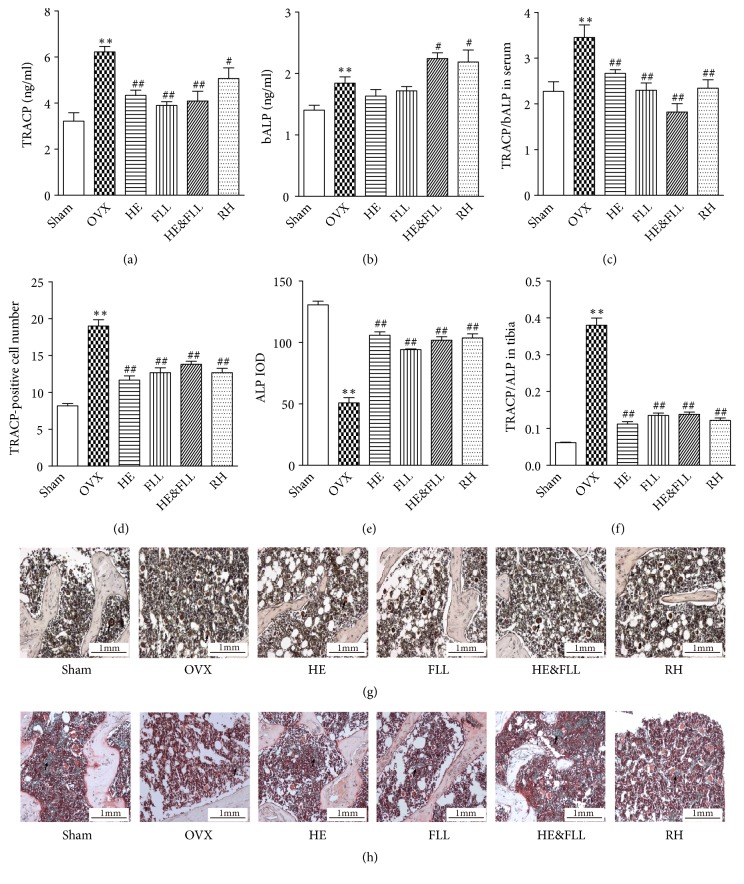
*Effects of HE & FLL on TRACP/ALP in OVX rats*. Serum TRACP (a) and bALP (b) were measured by ELISA. (c) TRACP/bALP in serum was calculated. Activity of TRACP (d) or ALP (e) in tibia was measured using ALP or TRACP stain kit. (f) TRACP/ALP in tibia was calculated. Representative photographs of TRACP (g) and ALP (h) staining were showed. Data are represented as mean ± SEM,* n* = 6. Note: ^*∗∗*^*P* < 0.01 versus Sham; ^#^*P* < 0.05 and ^##^*P* < 0.01 versus OVX.

ALP is a marker of osteogenesis, while TRACP is of bone resorption, so TRACP/ALP can be used to evaluate the balance of bone remodeling [14]. As shown in Figure 4, bALP and TRACP in serum and TRACP in bone were significantly increased after OVX, while ALP in bone was significantly decreased compared with Sham group (*P* < 0.01). Both TRACP/ALP in bone and serum were markedly increased after OVX compared with Sham group (*P* < 0.01). After treatment, rats in 4 treatment groups showed lower TRACP/ALP level compared with OVX group (*P* < 0.01), suggesting regulating effect on bone remodeling.

### 3.5. Effects of HE & FLL on Bone Resorption

**Figure 5:**
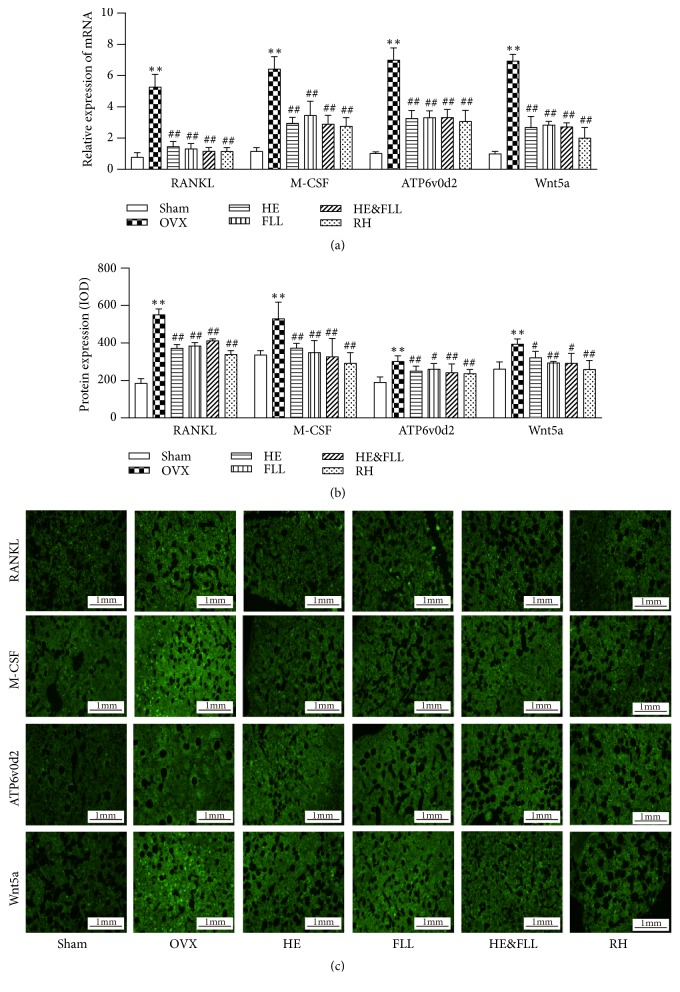
*Effects of HE & FLL on RANKL, M-CSF, ATP6v0d2, and Wnt5a in OVX rats*. (a) The mRNA expressions of RANKL, M-CSF, ATP6v0d2, and Wnt5a were measured by qRT-PCR analysis with *β*-actin as an internal control. (b) The protein expressions of RANKL, M-CSF, ATP6v0d2, and Wnt5a were measured by immunofluorescence staining. (c) Immunofluorescence images of RANKL, M-CSF, ATP6v0d2, and Wnt5a. Data are represented as mean ± SEM,* n* = 6. Note: ^*∗∗*^*P* < 0.01 versus Sham; ^#^*P* < 0.05 and ^##^*P* < 0.01 versus OVX.

RANKL, M-CSF, ATP6v0d2, and Wnt5a are important regulators associated with bone resorption. qRT-PCR analysis was carried out to evaluate their mRNA levels in femoral bone. The results indicated that significant increase in the mRNA expression of RANKL, M-CSF, ATP6v0d2, and Wnt5a were found in the OVX group compared to the Sham group (*P* < 0.01). And clear reduction in the mRNA levels of RANKL, M-CSF, ATP6v0d2, and Wnt5a was observed in all treatment groups when compared with the OVX rats (*P* < 0.01) (Figure 5(a)).

Simultaneously, we performed IF staining to determine the protein levels of RANKL, M-CSF, ATP6v0d2 and Wnt5a in rat femurs. In accordance with qRT-PCR results, compared to Sham group, the protein expression of RANKL, M-CSF, ATP6v0d2, and Wnt5a was significantly upregulated in OVX group (*P* < 0.01). All treatment groups could markedly downregulate RANKL, M-CSF, ATP6v0d2, and Wnt5a protein levels by comparing with OVX group (*P* < 0.05 or* P* < 0.01) (Figure 5(b)).

### 3.6. Effects of HE & FLL on Bone Formation

**Figure 6:**
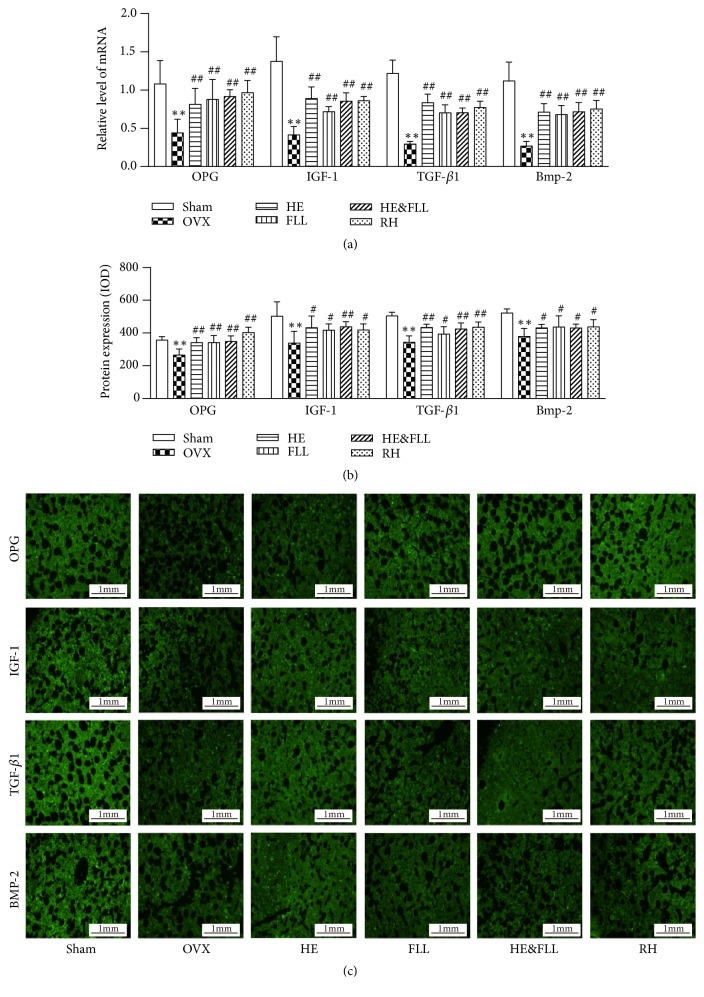
*Effects of HE & FLL on OPG, IGF-1, TGF-β1, and Bmp-2 in OVX rats*. (a) The mRNA expressions of OPG, IGF-1, TGF-*β*, and Bmp-2 were measured by qRT-PCR analysis with *β*-actin as an internal control. (b) The protein expressions of OPG, IGF-1, TGF-*β*1, and Bmp-2 were measured by immunofluorescence staining. (c) Immunofluorescence images of OPG, IGF-1, TGF-*β*1, and Bmp-2. Data are represented as mean ± SEM,* n* = 6. Note: ^*∗∗*^*P* < 0.01 versus Sham; ^#^*P* < 0.05 and ^##^*P* < 0.01 versus OVX.

IGF-1, TGF-*β*1, and Bmp-2 are important regulators associated with bone formation, and OPG can suppress the bone resorption by the inhibition of RANKL-RANK signaling. Contrary to bone resorption results, qRT-RCP analysis and IF staining of bone formation markers indicated significant decrease in both mRNA and protein expression of OPG, IGF-1, TGF-*β*1, and Bmp-2 in the OVX group compared to the Sham group (*P* < 0.01). And obvious increase in mRNA and protein levels of OPG, IGF-1, TGF-*β*1, and Bmp-2 was found in all treatment groups when compared with the OVX group (*P* < 0.05 or* P* < 0.01) (Figure 6).

## 4. Discussion

According to the TCM theories, “bone atrophy” (OP) is mainly caused by kidney deficiency. Therefore, the treatment of OP should follow the therapy of strengthening kidney function and restoring the balance between kidney-yin and kidney-yang. In China, HE and FLL, as kidney-tonifying Chinese herbal medicines, have been two thousand years old to treat orthopedic disease. A famous TCM doctor* Shizeng Li* has used HE and FLL to treat OP for more than 50 years. We had already attested the effects of combined extracts of HE and FLL (HE & FLL) on retinoic acid-induced OP rats and GIOP rats [4, 5, 13]. On this study, we wanted to investigate whether the combined herbs could treat PMOP.

OVX-induced OP, as a representative model for PMOP characterized by high bone remodeling, causes decreased bone mass and bone strength and increased fracture risk [19]. The main diagnostic criterion for OP is BMD, so we use this test to assess bone mass [20]. Meanwhile, bone biomechanics as the main parameter for predicting fractures had also been tested, including maximum load and ultimate displacement. We found in the experimental results that the levels of BMD, maximal load, and ultimate deflection were significantly decreased in OVX rats, indicating they have already suffered from OP. With the treatment of HE & FLL for 12 weeks, BMD and bone maximal load were improved. These results indicated that combined extracts of HE and FLL could play antiosteoporosis effects by improving bone mass and strengthen hardness and tenacity of bone at a lower dose.

Degeneration of cancellous bone is a typical pathological manifestation of OP. The degradation of the bone microstructure caused by OP is mainly reflected in the decrease of trabecular number and the increase in the trabecular separation, which destroys the connectivity of the trabecular bone [21]. Micro-CT, a nondestructive 3D imaging technology, is considered the most direct-viewing and effective method for assessing the structure and function of the bone [22]. In vivo micro-CT studies confirmed that OVX caused dynamic bone microstructure degradation on rats [23]. The results suggested that the rats in OVX group showed looser and intermittent trabecular bone comparing with rats in Sham group; the levels of BV/TV, Tb.Th, and Tb.N were obviously decreased, while BS/BV and Tb.Sp were markedly increased. After the treatment of HE & FLL, the rats showed serried and thicker bone trabeculas indicating significant effects on improving the microstructure of trabecular bone. However, due to the relatively short period of this study, the effect on cortical bone changes in OVX rats was not observed. Our results suggested that HE & FLL could improve bone mass and bone strength, reverse bone microstructure degradation, and reduce the risk of fracture.

Generally speaking, PMOP is mainly caused by estrogen deficiency. In our study, OVX rats showed a significant decrease in E_2_ levels, while HE & FLL could increase the levels of serum E_2_ in OVX rats. This result suggested that HE & FLL could show an antiosteoporosis effect by increasing estrogen levels. Estrogen deficiency which is associated with the imbalance between bone resorption capacity and bone formation ability leads to more bone cavities [24]. ALP is a highly sensitive marker of bone formation and is secreted by OBs [25]; TRACP is produced by OCs as a representative of bone resorption capacity [26]. In our present study, the number and activity of OCs were markedly increased in OVX rats, which were demonstrated by increased TRACP in bone and serum suggesting the increased bone resorption activity. Meantime, increased bALP in serum might demonstrate that the activity of bone-forming OBs was enhanced in OVX rats. Paradoxically, ALP expression in bone tissue was decreased. We thought the decreased bone formation was the results of OB apoptosis induced by estrogen deficiency [27, 28]. We calculated the ratio of TRACP to ALP to evaluate the bone remodeling rate. The results showed that TRACP/ALP was significantly elevated in OVX group, in both serum and bone tissue compared with Sham group, confirming that bone resorption exceeded formation. On the contrary, the levels of TRACP/ALP were markedly decreased after the treatment of HE & FLL, whether in serum or bone tissue. These results demonstrated the protective action of HE & FLL was related to maintaining the balance of bone remolding in OVX rats.

To further confirm the mechanism by which the two herbs maintain bone remolding balance, we detected the important bone turnover markers in OVX rats. Differentiation of mesenchymal stem cells into OC has two essential conditions: M-CSF and RANKL need to be combined with their receptor activators, respectively [29]. Numerous studies had shown that Wnt5a secreted by OB contributed to the production of OC, which enhances the expression of RANK, a receptor for RANKL, in precursor cells by activating the JNK/c-Jun signaling pathway [30]. Atp6v0d2, as a regulator of OCs fusion and bone formation, is possible to simultaneously promote OCs appreciation and inhabit OB function [31]. In this experiment, we observed the effects of HE & FLL on bone resorption by mRNA and protein expressions of RANKL, M-CSF, Wnt5a, and Atp6v0d2. As shown in results, the bone resorption activity was significantly enhanced in OVX rats, which was demonstrated by increased mRNA and protein expressions of bone resorption markers (RANKL, M-CSF, Wnt5a, and Atp6v0d2). Therefore, more bone resorption lacunas would appear because of hyperfunctional OCs. HE & FLL could markedly decrease the mRNA and protein expression of these bone resorption markers so as to inhibit bone resorption.

OPG, secreted by OBs, is the endogenous decoy receptor of RANKL with the function of inhibiting OC activation and promoting OC apoptosis [32]. IGF-1 is widely present in the adult body and is essential as an important metabolic hormone in regulating bone balance and maintaining bone physiological functions [33]. TGF-*β*1 plays an important role in regulating OB growth, differentiation, and immunologic function and Bmp-2 as a member of TGF-*β* superfamily can enhance bone formation by regulating osteoblastogenesis [34, 35]. We detected the above four bone formation markers to evaluate bone formation. The bone formation activity was decreased in OVX rats, while OVX rats treated with HE & FLL could significantly improve bone formation activity. By combining bone resorption markers, bone formation markers, and changes in TRACP/ALP, we inferred that HE & FLL rebalanced bone remolding by promoting osteoblastogenesis and inhibiting bone resorption simultaneously.

## 5. Conclusions

In summary, OVX-induced decrease in bone density, increased bone fragility, and deterioration of bone microstructure could be alleviated by the combined extracts of Herba Epimedii and Fructus Ligustri Lucidi, which increases bone mass while moderately reducing bone resorption. The combined extracts had antiosteoporosis effects through rebalance of bone remodeling on OVX rats, and they could be a reasonable agent for the treatment of PMOP.

## Figures and Tables

**Table 1:** Primer used for qRT-PCR.

Primer	Upstream	Downstream	Product size
RANKL	GCAGCATCGCTCTGTTCCTGTA	GCATGAGTCAGGTAGTGCTTCTGTG	164 bp
M-CSF	GAATGACTGAACCTGCCTGCTGAA	AGGCCAGCTCAGTGCAAGAA	117 bp
Wnt5a	ACAGGCAGTGGCATGCAGA	CAGGCAGCTGTTGACCTAGGAA	134 bp
Atp6v0d2	CGAGGCATTCTACAAATTCTGCAA	TTCAGTGCCAAATGAGTTCAGAGTG	124 bp
OPG	CTCATCAGTTGGTGGGAATGAAGA	ACCTGGCAGCTTTGCACAATTA	107 bp
IGF-1	GCACTCTGCTTGCTCACCTTTA	TCCGAATGCTGGAGCCATA	148 bp
TGF-*β*1	CATTGCTGTCCCGTGCAGA	AGGTAACGCCAGGAATTGTTGCTA	103 bp
Bmp-2	ACCGTGCTCAGCTTCCATCAC	CTATTTCCCAAAGCTTCCTGCATTT	170 bp
*β*-actin	CACTTTCTACAATGAGCTGCG	CTGGATGGCTACGTACATGG	129 bp

## Data Availability

The data used to support the findings of this study are available from the corresponding author upon request.
